# MicroRNA-21-3p modulates FGF2 to facilitate influenza A virus H5N1 replication by refraining type I interferon response

**DOI:** 10.1042/BSR20200158

**Published:** 2020-05-28

**Authors:** Jianli Shi, Ping Feng, Tingting Gu

**Affiliations:** Department of Paediatrics, The Fourth People’s Hospital of Jinan, Jinan 250031, Shandong, China

**Keywords:** FGF2, H5N1, miR-21-3p, replication, type I IFN response

## Abstract

**Background:** Influenza A virus (IAV) has greatly affected public health in recent decades. Accumulating data indicated that host microRNAs (miRNAs) were related to IAV replication. The present study mainly focused on the effects of microRNA-21-3p (miR-21-3p) on H5N1 replication.

**Methods:** The levels of miR-21-3p, virus structural factors (matrix 1 (M1), nucleoprotein (NP)), type I interferon (IFN) response markers (IFN-β, IFN-α), IFN-stimulated genes (protein kinase R (PKR), myxovirus resistance A (MxA), 2′-5′-oligoadenylate synthetase 2 (OAS)), and fibroblast growth factor 2 (FGF2) were measured by quantitative real-time polymerase chain reaction (qRT-PCR). The protein levels of M1, NP, and FGF2 were tested by Western blot assay. The virus titer was assessed by tissue culture infective dose 50% (TCID_50_) assay. The dual-luciferase reporter assay and ribonucleic acid (RNA) immunoprecipitation (RIP) assay were used to verify the interaction between miR-21-3p and FGF2.

**Results:** MiR-21-3p was reduced in H5N1-infected patients and A549 cells. MiR-21-3p overexpression facilitated the levels of M1, NP, TCID_50_ value, and reduced the levels of IFN-β, IFN-α, PKR, MxA, and OAS in H5N1-infected A549 cells. FGF2 was verified as a direct target of miR-21-3p. The introduction of FGF2 counteracted miR-21-3p-mediated decrease in the levels of M1, NP, and TCID_50_ value, as well as reduction in the levels of IFN-β, IFN-α, PKR, MxA, and OAS in H5N1-infected A549 cells.

**Conclusion:** MiR-21-3p down-regulated FGF2 expression to accelerate H5N1 replication and confine IFN response.

## Introduction

Influenza A virus (IAV) is a class of influenza virus, and the genome consists of eight negative-sense, single-stranded ribonucleic acid (RNA) [1]. Owing to the multiple combinations of 18 subtypes of H antigen and 11 subtypes of N antigen, IAV has many subtypes, such as H1N1, H5N1, and H7N9 [2]. In the past decades, IAV has been become a big threat to public health due to the characteristic of high variability [3]. Moreover, the researches about virus–host interactions and H5N1 infection has been extensively reported [4]. Therefore, it is crucial to explore the mechanism of H5N1 in influenza patients.

MicroRNAs (miRNAs), a type of small RNA (∼22 nucleotides (nts)) without translation capacity, affect the expression of target genes through inhibiting messenger RNA (mRNA) translation or degrading mRNA at transcriptional stage [5]. Emerging evidence indicated that dysregulation of miRNAs was involved in IAV replication. For example, a recent report implied that that overexpression of miR-33a restricted IAV infection by suppressing ARCN1 [6]. Another study demonstrated that the level of miR-485 was highly expressed in human airway epithelial cells infected with IAV, and its overexpression impeded the type I interferon (IFN) response and promoted virus replication [7]. Moreover, microRNA-21-3p (miR-21-3p) was reported to implicate in IAV replication [8]. However, the role of miR-21-3p in H5N1 replication and type I IFN response has been poorly documented.

Fibroblast growth factor 2 (FGF2), located on human chromosome 4q28.1, is implicated in many processes, including angiogenesis, embryonic development, and wound healing [9–11]. Also, FGF2 was pointed out to be associated with IAV replication [12]. However, the mechanism of FGF2 in H5N1 infection is still unclear. In the current exploration, the main research purpose was to elucidate the molecular mechanism of miR-21-3p in H5N1 infection in the host.

## Materials and methods

### Samples collection

Twenty-six H5N1-infected patients’ serum samples were obtained from H5N1-infected patients at The Fourth People’s Hospital of Jinan, as well as 13 serum samples from normal patients. The clinicopathological features of H5N1-infected patients were shown in Table 1. All serum samples were frozen at −80°C until further used. Written informed consents were provided by all patients. The study was permitted by the Ethics Committee of The Fourth People’s Hospital of Jinan and executed according to the Declaration of Helsinki Principles.

**Table 1:** Correlation between miR-21-3p level and clinicopathological features of H5N1-infected patients

Clinicopathologic features	Relative miR-21-3p level		*P*-value
	Low (%)	High (%)	
Gender (*n*)			*P*>0.05
Male	10 (58.8)	7 (41.2)	
Female	6 (54.5)	5 (45.5)	
Age (years)			*P*>0.05
≥30	9 (60.0)	6 (40.0)	
<30	7 (53.8)	6 (46.2)	
BMI			*P*>0.05
≥30	3 (60.0)	2 (40.0)	
<30	13 (56.5)	10 (43.5)	
Smoking history			*P*>0.05
Yes	7 (58.3)	5 (41.7)	
No	9 (56.3)	7 (43.7)	
Chronic lung disease			*P*<0.05
Yes	8 (88.9)	1 (11.1)	
No	8 (42.1)	11 (57.9)	
Chronic liver disease			*P*>0.05
Yes	3 (50.0)	3 (50.0)	
No	13 (59.1)	9 (40.9)	

### Cell culture and viruses

Human lung cancer cell line A549 was obtained from Procell (Wuhan, China). The cells were cultured in RPMI-1640 medium (Solarbio, Beijing, China) containing 10% fetal bovine serum (FBS; Solarbio) in an incubator with 5% CO_2_ at 37°C. H5N1 viruses A/Hong Kong/156/97 (HK/156) were done by serial passaging in specific-pathogen-free (SPF) 10-day-embryonated chicken eggs. All H5N1 experiments were carried out in a Biosafety Level 3 laboratory.

### Cell transfection and viral infection

MiR-21-3p mimics (mimic-21-3p) and its negative control (mimic-NC), miR-21-3p inhibitor (inhibitor-21-3p), and the negative control (inhibitor-NC), small interfering RNA (siRNA) against FGF2 (si-FGF2) and its matched control (si-NC) were bought from Sangon Biotech (Shanghai, China). The fragment of FGF2 was inserted into pcDNA vector (GenePharma, Shanghai, China) to construct overexpression plasmid (FGF2). The Lip2000 Transfection Reagent (Solarbio) was utilized for transfection. For viral infection, after transfection for 24 h, the washed A549 cells in the 24-well plate were infected with H5N1 at 0.1 multiplicity of infection (MOI) for 1 h. Then, the cells were incubated with fresh infection medium in an incubator.

### Quantitative real-time polymerase chain reaction

The RNA from A549 cells was extracted using TriQuick Reagent (Solarbio). Then, the reverse transcription for genes was carried out using AMV Reverse Transcriptase (Solarbio), while TaqMan miRNA assays (Applied Biosystems, Carlsbad, CA, U.S.A.) was used for miR-21-3p. The universal primer Uni-12 was used to reverse transcribe the influenza genome. The quantitative PCR was performed using SYBR Green PCR Master Mix (Ambion, Carlsbad, CA, U.S.A.). The relative expression level of miR-21-3p was normalized by small nuclear RNA U6, while genes were normalized via glyceraldehyde 3-phosphate dehydrogenase (GAPDH), and then calculated by the 2^−ΔΔ*C*_t_^ method. The primer sequences were shown in Table 2.

**Table 2:** Primers’ sequences

Name	Primer
miR-21-3p	F: ACACTCCAGCTGGGCAACACCAGTCGATGGGC
	R: CTCAACTGGTGTCGTGGA
NP	F: CCAGAAGCGGAGGAAACA
	R: GTCAAAGGAAGGCACGAT
M1	F: AAGACCAATCCTGTCACCTCTG
	R: CAAAACGTCTACGCTGCAGTCC
IFN-β	F: AGCTGAAGCAGTTCCAGAAG
	R: AGTCTCATTCCAGCCAGTGC
IFN-α	F: GTGAGGAAATACTTCCAAAGA
	R: TCTCATGATTTCTGCTCTGACA
PKR	F: TCTCAGCAGATACATCAGAGATAAATTCT
	R: AGTATACTTTGTTTCTTTCATGTCAGGAA
MxA	F: GGTGGTGGTCCCCAGTAATG
	R: ACCACGTCCACAACCTTGTCT
GAPDH	F: GAAGGTCGGAGTCAACGGATTT
	R: CTGGAAGATGGTGATGGGATTTC
OAS	F: GAAGCCCTACGAAGAATGTCAGA
	R: TCGGAGTTGCCTCTTAAGACTGT
U6	F: GCTTCGGCAGCACATATACTAAAAT
	R: CGCTTCACGAATTTGCGTGTCAT

### Western blot assay

The protein was extracted using RIPA solution (Solarbio), and its concentration was examined using a BCA kit (Beyotime, Shanghai, China). The equal amount of protein samples was separated by sodium dodecyl sulfonate/polyacrylamide gel electrophoresis (SDS/PAGE). Then, the protein was transferred onto a polyvinylidene fluoride (PVDF) membrane (GE Healthcare, Piscataway, NJ, U.S.A.). The membrane was first sealed into non-fat milk for 3 h at 37°C, followed by incubation with the primary antibodies overnight at 4°C and the secondary antibody for 4 h at 37°C. The chemiluminescence of bands was tested via an ECL kit (Beyotime). The primary antibodies against matrix protein 1 (M1), nucleoprotein (NP, 1/1000), FGF2 (1/1000), β-actin (1/10,00), and secondary antibody (1/10000) were bought from Bioss (Beijing, China).

### Tissue culture infective dose 50% assay

Virus titers were determined by tissue culture infective dose 50% (TCID_50_) assay. Briefly, A549 cells (8 × 10^3^ per well) samples were added to a 96-well plate and incubated for 16 h. Then, the diluted ten-fold gradient H5N1 samples were inoculated into each well and maintained for 96 h. Virus titers were estimated by the Reed–Muench method.

### Dual-luciferase reporter assay

The interaction between miR-21-3p and FGF2 was predicted by DIANA TOOLS (http://diana.imis.athena-innovation.gr). The wild-type (WT) and mutant (MUT) sequences of 3′-untranslated regions (3′UTRs) of FGF3 were inserted into pmirGLO vector (Youbio, Changsha, China) to construct luciferase reporter, namely FGF2-WT or FGF2-MUT. Then, 400 ng of luciferase reporter, 50 ng of *Renilla* luciferase reporter plasmid (pRL-TK), and 50 nM of mimic-21-3p or mimic-NC was transfected into cells using Lip2000 Transfection Reagent. The luciferase activity was detected using Dual-Lucy Assay Kit (Solarbio). *Renilla* luciferase activity was used as the internal control for the normalization of firefly luciferase activity.

### RNA immunoprecipitation assay

Magna RNA immunoprecipitation kit (Millipore, Billerica, MA, U.S.A.) was used to analyze the binding specificity between miR-21-3p and FGF2. Briefly, A549 cells were lysed in RNA immunoprecipitation (RIP) lysis buffer. Subsequently, the cell lysate sample was incubated with RIP buffer containing magnetic beads coupled with anti-Ago2 antibody (Millipore) or anti-IgG (Millipore). Then, the mixture was incubated with protease K to decompose the protein content, followed by analysis with quantitative real-time polymerase chain reaction (qRT-PCR).

### Statistical analysis

GraphPad Prism 7 (GraphPad Inc., La Jolla, CA, U.S.A.) was used to process the experimental data which were repeated three times. The Student’s *t* test was used to compare the data between the two groups, and one-way analysis of variance (ANOVA) was utilized to analyze the comparison among multiple groups. *P*-value less than 0.05 was considered statistically significant.

## Results

### MiR-21-3p was significantly decreased in H5N1-infected patients’ serum and A549 cells

**Figure 1:**
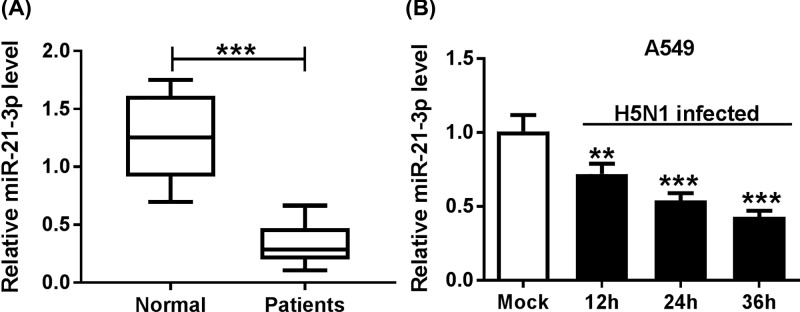
MiR-21-3p was significantly decreased in H5N1-infected patients’ serum and A549 cells (**A**) The level of miR-21-3p in patients infected with H5N1 (*n*=26) or normal patients (*n*=13) was examined by qRT-PCR. (**B**) The level of miR-21-3p in H5N1-infected A549 cells at different times or in negative control was tested via qRT-PCR. **P<0.01, ***P<0.001.

Recent studies indicated that miR-21-3p was implicated in IAV replication. Thus, we first detected the level of miR-21-3p in 26 serums of H5N1-infected patients. As exhibited in Figure 1A, the level of miR-21-3p was strikingly declined in patients infected with H5N1 compared with that in normal patients. Moreover, in contrast with cells infected with negative control, the level of miR-21-3p in A549 cells infected with H5N1 was conspicuously down-regulated with prolonged infection (Figure 1B). These data indicated that H5N1 infection reduced the level of miR-21-3p.

### MiR-21-3p promoted H5N1 replication in H5N1-infected A549 cells

**Figure 2:**
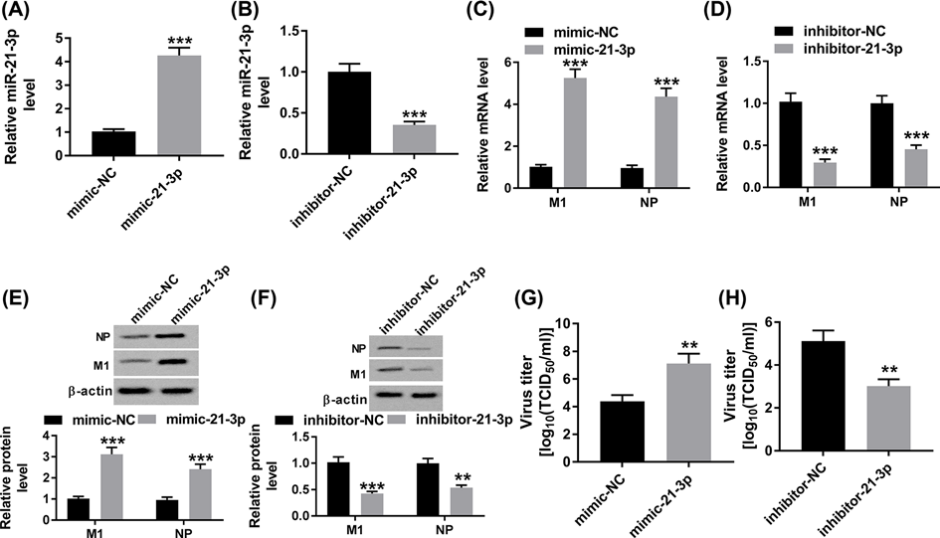
MiR-21-3p promoted H5N1 replication in H5N1-infected A549 cells (**A**–**H**) The H5N1-infected A549 cells were transfected with mimic-NC, mimic-21-3p, inhibitor-NC, or inhibitor-21-3p. (A,B) The level of miR-21-3p was measured by qRT-PCR. (C,D) The mRNA levels of M1 and NP were detected via qRT-PCR. (E,F) The protein levels of M1 and NP were evaluated by Western blot assay. (G,H) The virus titer was assessed by TCID_50_ assay. **P<0.01, ***P<0.001.

In order to explore the effect of miR-21-3p on H5N1 replication, mimic-21-3p, or inhibitor-21-3p was transfected into H5N1-infected A549 cells. qRT-PCR results confirmed the transfection efficiency, presenting as the apparent up-regulation of miR-21-3p in H5N1-infected A549 cells transfected with mimic-21-3p or the remarkable decline of miR-21-3p with inhibitor-21-3p (Figure 2A,B). Since M1 as a matrix protein and NP as a major NP [13,14], the mRNA and protein levels of M1 and NP were measured in H5N1-infected A549 cells transfected with mimic-21-3p or inhibitor-21-3p. As shown in Figure 2C–F, the mRNA and protein levels of M1 and NP were markedly increased in H5N1-infected A549 cells transfected with mimic-21-3p, while their levels were distinctly reduced in H5N1-infected A549 cells transfected with inhibitor-21-3p. Moreover, the TCID_50_ assay presented that the transfection of mimic-21-3p contributed to the obvious augment of infectious progeny virions in H5N1-infected A549 cells, but the infectious progeny virions were effectively decreased in inhibitor-21-3p group (Figure 2G,H). Taken together, miR-21-3p accelerated H5N1 replication in H5N1-infected A549 cells.

### MiR-21-3p suppressed the type I IFN response induced by H5N1 infection

**Figure 3:**
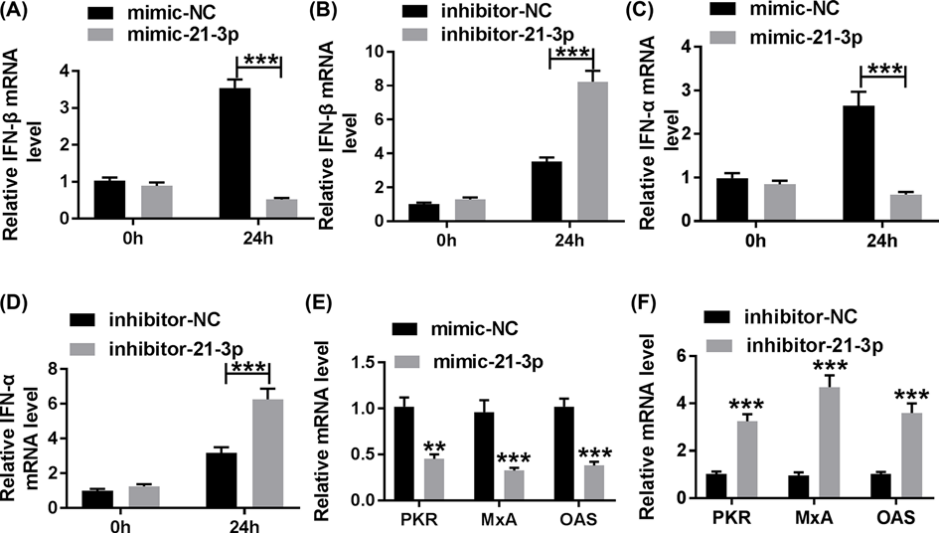
MiR-21-3p suppressed IFN response in H5N1-infected A549 cells (**A–F**) The H5N1-infected A549 cells were transfected with mimic-21-3p, mimic-NC, inhibitor-NC, or inhibitor-21-3p. (A,B) The level of IFN-β was measured via qRT-PCR in treated A549 cells. (C,D) The level of IFN-α in treated A549 cells was tested by qRT-PCR. (E,F) The levels of PKR, MxA, and OAS in treated A549 cells were assessed by qRT-PCR. **P<0.01, ***P<0.001.

To explore whether miR-21-3p was involved in the type I IFN response, the effects of miR-21-3p knockdown or overexpression on IFN factors were studied. Since IFN-β and IFN-α are IFN markers, the levels of IFN-β and IFN-α were measured in H5N1-infected A549 cells. As presented in Figure 3A–D, the levels of IFN-β and IFN-α were dramatically down-regulated in H5N1-infected A549 cells transfected with mimic-21-3p after 24-h infection, while silencing of miR-21-3p produced the opposite results. Given that protein kinase R (PKR), myxovirus resistance A (MxA), and 2′-5′-oligoadenylate synthetase (OAS) were IFN-stimulated genes, the levels of them were also detected in H5N1-infected A549 cells. The trends of PKR, MxA, and OAS were similar with IFN-β and IFN-α. In brief, the introduction of mimic-21-3p notably reduced the levels of PKR, MxA, and OAS in H5N1-infected A549 cells, while the levels of PKR, MxA, and OAS in inhibitor-21-3p group were prominently elevated (Figure 3E,F). To sum up, miR-21-3p refrained type I IFN response induced by H5N1 infection.

### MiR-21-3p bound to FGF2 in H5N1-infected A549 cells

**Figure 4:**
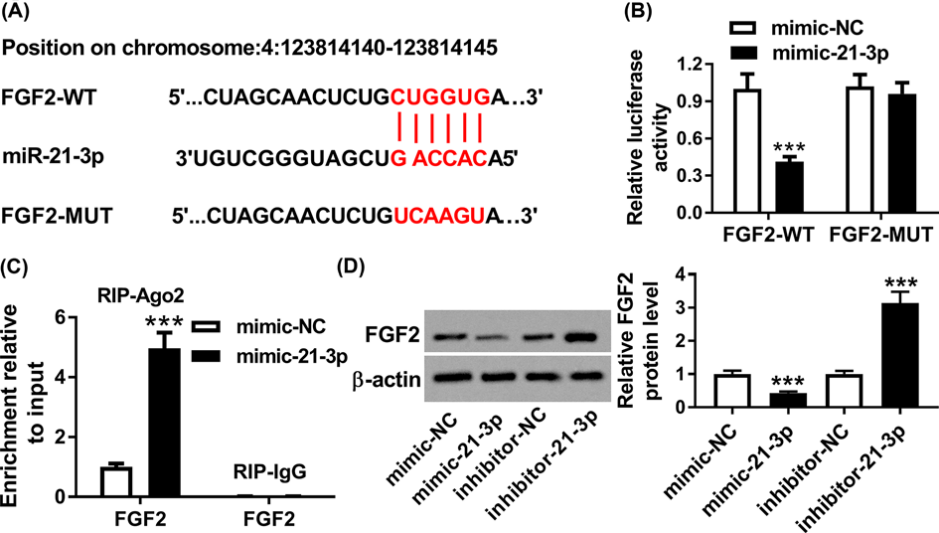
FGF2 was a target of miR-21-3p in H5N1-infected A549 cells (**A**) The complementary binding sites between FGF2 3′UTR and miR-21-3p were presented, as well as the mutant sequences of FGF2. (**B**) The luciferase activity of FGF2-WT or FGF2-MUT reporter in H5N1-infected A549 cells transfected mimic-NC or mimic-21-3p was evaluated by dual-luciferase reporter assay. (**C**) The enrichment of FGF2 was tested via RIP assay. (**D**) The protein level of FGF2 in H5N1-infected A549 cells transfected with mimic-NC, mimic-21-3p, inhibitor-NC, or inhibitor-21-3p was detected by Western blot assay. ***P<0.001.

To illustrate the mechanism of miR-21-3p in H5N1 replication, DIANA TOOLS online database was utilized to predict the potential target of miR-21-3p. As exhibited in Figure 4A, FGF2 3′UTR had complementary base pairing with miR-21-3p. The following dual-luciferase reporter assay indicated that the introduction of mimic-21-3p resulted in the striking decline of luciferase activity of FGF2-WT reporter in H5N1-infected A549 cells in comparison with that in mimic-NC group, while the luciferase activity of FGF2-MUT reporter had no obvious fluctuation in any group (Figure 4B). Moreover, RIP assay suggested that the Ago2 enriched much more FGF2 in H5N1-infected A549 cells transfected with mimic-21-3p related to that in IgG group (Figure 4C). In addition, Western blot assay showed that the protein level of FGF2 was significantly decreased in H5N1-infected A549 cells transfected with mimic-21-3p, but FGF2 level was remarkably enhanced in inhibitor-21-3p group (Figure 4D). These results implied that FGF2 was a direct target of miR-21-3p in H5N1-infected A549 cells.

### MiR-21-3p promoted H5N1 replication in H5N1-infetced A549 cells by down-regulating FGF2

**Figure 5:**
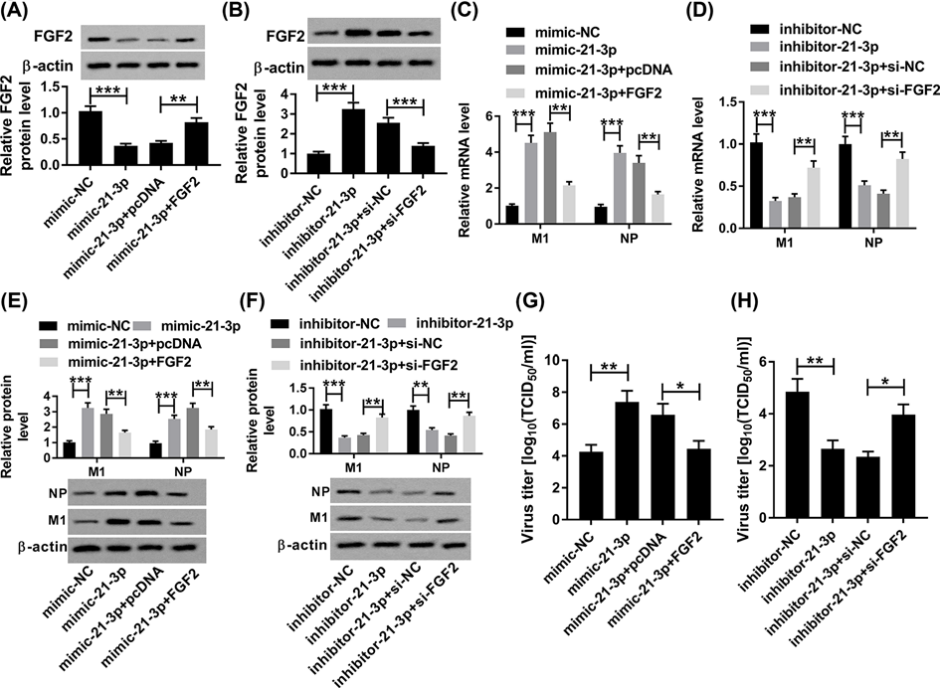
MiR-21-3p promoted H5N1 replication caused by H5N1 infection (**A,C,E,G**) The H5N1-infected A549 cells were transfected with mimic-NC, mimic-21-3p, mimic-21-3p + pcDNA, or mimic-21-3p + FGF2. (**B,D,F,H**) The H5N1-infected A549 cells were transfected with inhibitor-NC, inhibitor-21-3p, inhibitor-21-3p + si-NC, or inhibitor-21-3p + si-FGF2. (A,B) The protein level of FGF2 was tested via Western blot assay. (C,D) The levels of M1 and NP were measured by qRT-PCR. (E,F) The protein levels of M1 and NP were examined via Western blot assay. (G,H) The virus titer was assessed by TCID_50_ assay. *P<0.05, **P<0.01, ***P<0.001.

To further elucidate whether the promotion of miR-21-3p to H5N1 replication was mediated by FGF2, the effects of miR-21-3p and FGF2 were studied together. First, Western blot results suggested that the protein level of FGF2 was dramatically decreased in mimic-21-3p group, which was partly regained by the re-introduction of FGF2 (Figure 5A). However, the level of FGF2 protein showed the opposite trends in inhibitor-21-3p and si-FGF2 group. Briefly, the promotion effect on the protein level of FGF2 caused by down-regulation of miR-21-3p was mitigated by FGF2 knockdown (Figure 5B). Furthermore, the transfection of FGF2 attenuated the facilitated impacts on the mRNA and protein levels of M1 and NP, as well as the infectious progeny virions in H5N1-infected A549 cells caused by mimic-21-3p; whereas these showed the contrary trends in inhibitor-21-3p and si-FGF2 group (Figure 5C–H). Taken together, these data revealed that miR-21-3p aggravated H5N1 replication in H5N1-infetced A549 cells by down-regulating FGF2.

### MiR-21-3p confined IFN response in H5N1-infected A549 cells by down-regulating FGF2

**Figure 6:**
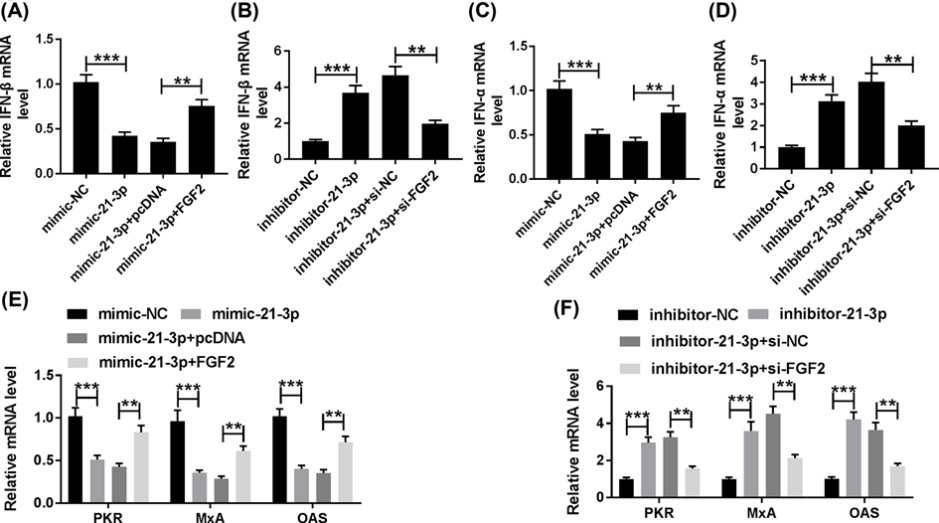
MiR-21-3p confined IFN response in H5N1-infected A549 cells by modulating FGF2 (**A,C,E**) The H5N1-infected A549 cells were transfected with mimic-NC, mimic-21-3p, mimic-21-3p + pcDNA, or mimic-21-3p + FGF2. (**B,D,F**) The H5N1-infected A549 cells were transfected with inhibitor-NC, inhibitor-21-3p, inhibitor-21-3p + si-NC, or inhibitor-21-3p + si-FGF2. (A,B) The level of IFN-β was assessed via qRT-PCR. (C,D) The level of IFN-α was measured by qRT-PCR. (E,F) The levels of PKR, MxA, and OAS were detected via qRT-PCR. **P<0.01, ***P<0.001.

Also, we explored the effects of miR-21-3p and FGF2 on IFN response. The emergence of FGF2 relieved the suppressive effect on the levels of IFN-β, IFN-α, PKR, MxA, and OAS in H5N1-infected A549 cells refrained by mimic-21-3p (Figure 6A,C,E). Nevertheless, the levels of IFN-β, IFN-α, PKR, MxA, and OAS showed the inverse tendency in inhibitor-21-3p and si-FGF2 group. Briefly, the levels of IFN-β, IFN-α, PKR, MxA, and OAS were first augmented in inhibitor-21-3p group, while the re-introduction of si-FGF2 overturned this accelerated impact (Figure 6B,D,F). To sum up, miR-21-3p down-regulated FGF2 expression to retard type I IFN response in H5N1-infected A549 cells.

## Discussion

In the recent several years, H5N1, a highly pathogenic avian influenza, has affected the lives of many people, even life-threatening. Emerging data indicated that miRNAs were involved in host–virus interactions [15]. This exploration was concentrated on the molecular mechanism of miR-21-3p on H5N1 replication *in vitro*. A549 cells are a kind of human lung cancer cells that have been used as models for IAV infection *in vitro* due to the sensitivity to IAV infection [16]. These data disclosed that miR-21-3p down-regulated FGF2 expression to facilitate H5N1 replication and confine type I IFN response.

Recent studies revealed that host miRNAs were associated with IAV replication. For example, Zhao et al. reported that miR-340-5p was reduced in IAV-infected A549 cells, and its overexpression facilitated IAV replication through RIG-I signaling [17]. Conversely, another report presented that miR-203 was enhanced following IAV infection, and its overexpression restrained IAV replication by regulating DR1 [18]. The molecular processes of IAV infection were a complex network, and miRNAs played different roles in this network. In the current study, miR-21-3p was decreased in H5N1-infected patients and A549 cells. Moreover, overexpression of miRNA-21-3p elevated the production of M1 and NP, as well as the TCID_50_ value, in accordance with previous report [8]. These data unraveled that miR-21-3p facilitated H5N1 replication.

Accumulating evidence demonstrated that dysregulation of host miRNAs affected the type I IFN response. For instance, a document indicated that miR-146a overexpression refrained IFN response during IAV infection [19]. Consistently, another report showed that miR-302c overexpression curbed IFN response following IAV infection [20]. In this exploration, overexpression of miR-21-3p reduced the levels of IFN markers (IFN-β and IFN-α) and stimulated genes (PKR, MxA, and OAS). These data disclosed that miR-21-3p impeded type I IFN response in H5N1 infection. Moreover, miRNAs was involved in NF-κB signaling pathway, and NF-κB is a production of type I IFN response [21–23]. Thus, we will further investigate the role of miR-21-3p in NF-κB signaling pathway in H5N1 infection in the future study.

Convincing data disclosed that the aberrant expression of FGF2 was implicated in the molecular processes of IAV infection. For example, Wang et al. documented that FGF2 was elevated in H1N1-infected patients, and its overexpression mitigated the IAV-induced injury [24]. Another research reported that FGF2 repressed H1N1 replication by enhancing type I IFN production regulated by miR-194 [12]. In the present research, FGF2 was validated as a candidate target of miR-21-3p. We hypothesized that FGF2 might be increased in H5N1-infected patients and A549 cells. Functionally, FGF2 silencing regained the constraint impacts on the levels of M1 and NP and the TCID_50_ value, as well as the promotion effect on the levels of IFN-β, IFN-α, PKR, MxA, and OAS in H5N1-infected A549 cells retarded by miR-21-3p inhibitor. These data demonstrated that miR-21-3p down-regulated FGF2 to accelerate H5N1 replication through impeding the type I IFN response.

In conclusion, miR-21-3p was reduced in patients and A549 cells infected with H5N1. MiR-21-3p targeted FGF2 to accelerate H5N1 replication in H5N1-infected A549 cells by inhibiting type I IFN response. The miR-21-39/FGF2 axis in H5N1 infection may provide the experimental foundation for the exploration of host–virus interactions in the future.

## Abbreviations

FGF2: fibroblast growth factor 2
IAV: influenza A virus
IFN: interferon
miRNA: microRNA
miR-21-3p: microRNA-21-3p
mRNA: messenger RNA
MxA: myxovirus resistance A
M1: matrix 1
NP: nucleoprotein
OAS: 2′-5′-oligoadenylate synthetase
PKR: protein kinase R
qRT-PCR: quantitative real-time polymerase chain reaction
RIP: RNA immunoprecipitation
RNA: ribonucleic acid
TCID_50_: tissue culture infective dose 50%
3′UTR: 3′-untranslated region
